# Individual and shared effects of social environment and polygenic risk scores on adolescent body mass index

**DOI:** 10.1038/s41598-018-24774-5

**Published:** 2018-04-20

**Authors:** Jonathan R. I. Coleman, Eva Krapohl, Thalia C. Eley, Gerome Breen

**Affiliations:** 1 King’s College London, Institute of Psychiatry, Psychology and Neuroscience, Social, Genetic and Developmental Psychiatry (SGDP) Centre, London, UK; 2 National Institute for Health Research Biomedical Research Centre, South London and Maudsley National Health Service Trust, London, UK

## Abstract

Juvenile obesity is associated with adverse health outcomes. Understanding genetic and environmental influences on body mass index (BMI) during adolescence could inform interventions. We investigated independent and interactive effects of parenting, socioeconomic status (SES) and polygenic risk on BMI pre-adolescence, and on the rate of change in BMI across adolescence. Genome-wide genotype data, BMI and child perceptions of parental warmth and punitive discipline were available at 11 years old, and parental SES was available from birth on 3,414 unrelated participants. Linear models were used to test the effects of social environment and polygenic risk on pre-adolescent BMI. Change in BMI across adolescence was assessed in a subset (N = 1943). Sex-specific effects were assessed. Higher genetic risk was associated with increased BMI pre-adolescence and across adolescence (p < 0.00417, corrected for multiple tests). Negative parenting was not significantly associated with either phenotype, but lower SES was associated with increased BMI pre-adolescence. No interactions passed correction for multiple testing. Polygenic risk scores from adult GWAS meta-analyses are associated with BMI in juveniles, suggesting a stable genetic component. Pre-adolescent BMI was associated with social environment, but parental style has, at most, a small effect.

## Introduction

The prevalence of overweight and obesity is increasing in children and adolescents in developed countries, such that over 20% of individuals under the age of nineteen have a body mass index (BMI) > 25^1^. High BMI in this period is associated with psychosocial discrimination, and with socioeconomic hardship and increased cardio-metabolic morbidity in later life^2,3^. Understanding the aetiology of juvenile BMI and of factors influencing change in BMI across adolescence could be informative in developing interventions, and alleviating current and future personal and economic costs^4,5^.

There is robust evidence that variation in BMI is influenced by genetic factors, both from studies of rare variants (such as perturbations in the leptin signalling pathway) and from large genome-wide association studies in adults and in children^6–9^. Furthermore, there is evidence for a stable genetic influence on BMI, with genetic variants associated with BMI in adulthood predictive of weight gain in early childhood^10^, across childhood and adolescence^11^, and throughout the life-course^12^. Evidence from both neuroendocrinological and statistical genetic approaches suggest brain expressed genes may underlie variation in BMI, potentially through controlling energy homeostasis directly within the body as well as via behavioural processes such as eating and exercise^4,13^.

The rapid increase in obesity in the last three decades argues for a role of environmental factors, potentially acting to mediate genetic predispositions^3,4^. Parenting is one factor that can influence childhood BMI directly through diet and via learnt food-related behaviours in children, including dietary self-control and regulation of active and sedentary behaviours^14^. However, excessive parental control over food intake behaviours can have a rebound effect when that control is relaxed, such that children over-indulge in previously restricted foodstuffs^15^. Much of the research on parenting style and BMI has focussed on the related concepts of parental control and involvement, with some evidence suggesting a controlled disciplinary style and positive parent-child interactions are associated with greater control over BMI levels in childhood^16^.

Parenting style represents one part of the wider influence of socioeconomic environment on child development^14^. Broader measures, such as parental socioeconomic status (SES), may capture this more general influence. In the particular case of BMI, low SES has been associated with higher BMI, particularly in adolescents and young adults^17–19^. However, reported results vary according to gender, ethnicity and nationality, and there is a potential cohort effect, with null results more common in cohorts ascertained less recently^20–22^.

There is an observable difference in BMI pre-adolescence between females and males, due in part to the earlier onset of puberty in females, and there is an ongoing debate whether the genetic aetiology of pre-adolescent BMI is sex-specific^23,24^. A combined analysis of twin studies examining BMI in pre-adolescence did not identify any difference in heritability between sexes, but lacked necessary data (such as opposite sex dizygotic twin pairs) to make strong conclusions^24^. In contrast, a larger, multi-national study of young adult twin pairs found higher heritability for BMI in females, with results largely consistent across national studies^23^.

Evidence that genetic and environmental influences contribute to BMI has prompted a considerable number of studies exploring gene-by-environment interactions^25^. Of these, the interaction between variation in the *FTO* gene and physical activity is the most robust, although the functional mechanism of this interaction remains an area of active research^26,27^. Beyond this interaction, most studies have explored the effects of single variants in the context of many different environments^25^. However, this approach has been limited due to small sample sizes (and hence low power), inadequate sampling of variation at the genetic locus of interest, and a potentially incorrect hypothesis-driven approach^28^. Recent studies have begun to address this criticism by using gene scores that include associated variants from genome-wide meta-analyses of BMI^29^. This technique can be extended by using weighted polygenic risk scores, which use genome-wide genotypes to construct scores, weighting each variant (commonly by its effect size in genome-wide association study meta-analyses^30^).

We investigated the independent and interactive effects of social environmental variables and genetic influences on BMI pre-adolescence, and on the rate of change in BMI across adolescence, in a cohort of unrelated adolescents representative of the population of the United Kingdom (the Twins Early Development Study: TEDS^31–33^). The contribution of genetic factors to phenotypic variance was estimated using the most associated polygenic risk score from the largest genome-wide association study meta-analysis in BMI published to date^6^. During these analyses, it became apparent that SES contributes to the aetiology of BMI in a manner that overlaps with the effect of parental warmth and punitive discipline. As such, secondary analyses were performed assessing the effect of SES in the place of parenting.

## Results

### Demographics

Demographic data on the analysed cohort and subsets are available in Table 1. On average, the subset of the cohort in which change in BMI across adolescence was assessed was significantly older and had progressed further into adolescence at the baseline assessment than the cohort as a whole. Although SES was higher in the subset, the difference was not significant after multiple testing (Welch two sample t-test, Bonferroni correction for 6 tests, *p* = 0.0083; Table 1). Females were significantly more developed than males, reported less harsh and punitive parenting, and had higher BMI, although the difference in BMI was not significant in the subset with multiple BMI assessments (Table 2).

**Table 1:** Demographic data of the full cohort and subset in which change in BMI was studied.

Variable	Full Cohort	BMI Change Subset	t-test *p*
N	3414	1943	—
Female sex (%)	51.3	53.7	0.0881
**Age (mean**, **SD)**	**11**.**3 (0**.**695)**	**11**.**5 (0**.**616)**	**1**.**52 × 10**^**−27**^
SES at birth (mean, SD)	0.235 (0.961)	0.289 (0.958)	0.0474
**Pubertal development (mean**, **SD)**	**1**.**69 (0**.**554)**	**1**.**74 (0**.**567)**	**0**.**00310**
BMI (mean, SD)	17.8 (3.07)	17.9 (3.101)	0.188
Parenting style (mean, SD)	0 (1.71)	−0.0733 (1.70)	0.131
Change in BMI (mean, SD)	—	0.580 (0.0995)	—

Age and pubertal development were significantly higher in the subset after multiple testing correction; SES was greater in the subset, but did not pass multiple testing correction (Welch two sample t-test, *p* = 0.05/6 = 0.0083).

**Table 2:** Demographic data stratified by sex.

Variable	Full Cohort		t-test *p*
	Females	Males	
N	1750	1664	—
Age (mean, SD)	11.3 (0.693)	11.2 (0.697)	0.550
SES at birth (mean, SD)	0.232 (0.957)	0.238 (0.965)	0.862
**Pubertal development (mean**, **SD)**	**1**.**85 (0**.**589)**	**1**.**52 (0**.**457)**	**1**.**79 × 10**^**−72**^
**BMI (mean**, **SD)**	**17**.**9 (3**.**22)**	**17**.**6 (2**.**90)**	**0**.**00179**
**Parenting style (mean**, **SD)**	**−0**.**152 (1**.**69)**	**0**.**160 (1**.**73)**	**9**.**87 × 10**^**−8**^
**Variable**	**BMI Change Subset**		**t-test** ***p***
	**Females**	**Males**	
N	1043	900	—
Age (mean, SD)	11.4 (0.633)	11.5 (0.584)	0.126
SES at birth (mean, SD)	0.294 (0.947)	0.283 (0.970)	0.805
**Pubertal development (mean**, **SD)**	**1**.**90 (0**.**600)**	**1**.**56 (0**.**465)**	**1**.**06 × 10**^**−42**^
BMI (mean, SD)	18.0 (3.33)	17.8 (2.83)	0.206
**Parenting style (mean**, **SD)**	**−0**.**234 (1**.**67)**	**0**.**113 (1**.**72)**	**7**.**53 × 10**^**−6**^
Change in BMI (mean, SD)	0.581 (0.103)	0.580 (0.0951)	0.764

Demographic data of the full cohort and subset in which change in BMI was studied, stratified by sex. Females were significantly more developed and reported less harsh and punitive parenting, and exhibited high BMI (the last of which only in the full cohort; Welch two sample t-tests, *p* = 0.05/6 = 0.0083).

Correlations between variables included in the analyses are displayed in Supplementary Table S1. Genotyping wave was strongly correlated with the first principal component (r = 0.71), and BMI at 11 was strongly correlated with change in BMI across adulthood (r = −0.51). Repeating the analyses without including genotyping wave as a covariate did not alter the conclusions of the study. BMI at 11 is also strongly correlated with the random intercepts used in the construction of the change phenotype (r ≈ 0.9). As such, BMI at 11 is an integral part of the change phenotype and its inclusion is required for the proper interpretation of these analyses. No other strong correlations were observed (all |r| < 0.5).

### Polygenic risk scoring

Polygenic risk analyses identified a score comprised of 2321 independent variants with *p* ≤ 0.0032 in the GIANT 2015 all ancestries GWAS, which predicted a significant proportion of variance in BMI at 11 years of age (*p* = 1.77 × 10^−37^, R^2^ = 0.0469^6^). This is consistent with previous estimations of polygenic risk in the TEDS cohort at age 16^34^. In cross-trait analyses, scores from the GIANT GWAS were not associated with parenting (best threshold = 0.0845, N_SNPS_ = 18336, p = 0.0663, R^2^ = 9.89 × 10^−4^). Similar analyses with SES identified a significant association with the BMI polygenic score when it was optimised for SES (threshold = 0.0795, N_SNPS_ = 17580, p = 9.18 × 10^−5^, R^2^ = 0.00445), but not when it was optimised for BMI (threshold = 0.0032, N_SNPS_ = 2321, p = 0.154, R^2^ = 5.93 × 10^−4^). The score optimised for BMI is reported in all main analyses.

### BMI at 11

Higher genetic risk was associated with higher BMI at 11 years old (Table 3). A nominally significant effect of colder and more punitive parenting associated with higher BMI was observed, but was not significant after Bonferroni correction for twelve tests (*p* = 0.05/12 ≈ 0.00417). No interaction between risk and parenting was identified in the main analysis, or after stratifying by sex (Supplementary Table S2a). In secondary analyses with SES as the environment of interest, lower SES was associated with higher BMI. The effect of SES was largely independent of the effect of genetic risk; the inclusion of both variables in the model did not substantially alter the effect sizes observed when each variable was included alone. The interaction between SES and genetic risk was nominally significant, but did not survive correction for multiple testing. No sex-specific effects were observed (Supplementary Table S2b).

**Table 3:** Stepwise addition of variables to linear model predicting log(BMI) at 11 years old.

Coefficient	B	SE	*p*	Adjusted R^2^
**BMI at 11 years old**, **with parenting**				
Null model	Supplementary Table S2a			0.0667
(Null model) + Parental style	0.0378	0.0167	0.0239	0.0678
(Null model) + **BMI PRS**	**0**.**210**	**0**.**0162**	**1**.**59 × 10**^**−37**^	0.110
(Null model) + Parental style + **BMI PRS**	0.0360 **0**.**210**	0.0163 **0**.**0162**	0.0273 **1**.**83 × 10**^**−37**^	0.111
(Null model) + Parental style × BMI PRS	0.00642	0.0172	0.709	0.113
**BMI at 11 years old**, **with SES**				
Null model	Supplementary Table S2b			0.0628
Null model + **SES**	**−0**.**0729**	**0**.**0167**	**1**.**33 × 10**^**−5**^	0.0678
Null model + **BMI PRS**	**0**.**211**	**0**.**0162**	**7**.**95 × 10**^**−38**^	0.107
Null model + **SES** + **BMI PRS**	**−0**.**0682** **0**.**210**	**0**.**0163** **0**.**0162**	**3**.**11 × 10**^**−5**^ **1**.**83 × 10**^**−37**^	0.111
Null model + SES × BMI PRS	−0.0336	0.0165	0.0413	0.112

Effects of adding variables and interactions to the null model predicting variance in log(BMI) at 11 years old, with parenting (above) and SES (below) as the environment of interest. Significant (p < 0.00417) terms are in bold. Interactions include all main effects, covariates and covariate interaction terms^60^.

### Change in BMI during adolescence

Higher genetic risk was associated with a greater increase in BMI (Table 4). Genetic risk was significantly associated with change in BMI in females but not in males (Supplementary Table S3a). However, the interaction between PRS and sex was not significant in the main analysis (p = 0.240). No interaction between genetic risk and parental warmth and discipline was observed. In secondary analyses with SES as the environment of interest, there was no significant main effect of SES. The interaction between genetic risk and SES was nominally significant when both sexes were analysed together and in females only (Supplementary Table S3b). However, neither the interaction in the full analysis nor that in the female-only subset was significant after correction for multiple testing.

**Table 4:** Stepwise addition of variables to linear model predicting change in log(BMI) between 11-16 years

Coefficient	B	SE	*p*	Adjusted R^2^
**Change in BMI**, **with parenting**				
Null model	Supplementary Table S3a			0.277
(Null model) + Parental style	−0.00848	0.0196	0.666	0.277
(Null model) + **BMI PRS**	**0**.**0902**	**0**.**0197**	**4**.**96 × 10**^**−6**^	0.284
(Null model) + Parental style + **BMI PRS**	−0.00969 **0**.**0903**	0.0195 **0**.**0197**	0.620 **4**.**84 × 10**^**−6**^	0.284
(Null model) + Parental style × BMI PRS	0.000551	0.0207	0.979	0.285
**Change in BMI**, **with SES**				
Null model	Supplementary Table S3b			0.277
Null model + SES	0.0106	0.0196	0.591	0.277
Null model + **BMI PRS**	**0**.**0904**	**0**.**0197**	**4**.**70 × 10**^**−6**^	0.284
Null model + SES + **BMI PRS**	0.00965 **0**.**0903**	0.0195 **0**.**0197**	0.622 **4**.**84 × 10**^**−6**^	0.284
Null model + SES × BMI PRS	−0.0494	0.0205	0.0159	0.289

Effects of adding variables and interactions to the null model (uppermost line) predicting variance in change in log(BMI) across adolescence, with parenting(above) and SES (below) as the environment of interest. Significant (p < 0.00417) terms are in bold. Interactions include all main effects, covariates and covariate interaction terms^60^.

### Power

Post-hoc power calculations suggested that the full sample was powered to detect small effects (80% power to detect Cohen’s f^2^ = 0.00229 at age 11, f^2^ = 0.00410 for change in BMI), as were sex-stratified analyses (BMI at age 11: f^2^ = 0.00452 and f^2^ = 0.00475; change in BMI across adolescence: f^2^ = 0.00806 and f^2^ = 0.00832 for females and males respectively). For context, Cohen suggested f^2^ = 0.02 as a small effect^35^.

### Sensitivity analyses

Conclusions from sensitivity analysis did not differ from those drawn from the main analysis (Supplementary Material).

## Discussion

### Summary of findings

This study examined the relationship between genetic and social environmental effects (individually and in combination) and two BMI phenotypes: BMI prior to adolescence and the rate of change in BMI between 11 and 16. Genetic effects associated with higher BMI in the largest cohort published to date (the 2015 GIANT consortium meta-analysis) were associated with higher BMI before adolescence, and with a greater increase in BMI across adolescence^6^. In contrast, child perceptions of parental warmth and discipline were not significantly associated with pre-adolescent BMI or with change in BMI across adolescence in this study. However, lower parental SES, as a more general measure of childhood social environment, was associated with higher BMI pre-adolescence, but not with change in BMI.

### Limitations

The measures used in this study are unlikely to capture the full component of variance they each represent. The PRS is limited to the effects of common variants on BMI in an additive model, and only to those regions of the genome that are captured adequately by both the GIANT BMI GWAS and the TEDS study genotyping (Supplementary Material^6^). In addition, only a small proportion of the genetic component of variance in BMI was captured by the PRS in this study (7–14%, assuming a heritability of BMI of 30–60%^36,37^). Finally, these analyses used the optimal PRS (that is, the one explaining the most variance in BMI as a main effect). Multiple PRS, generated using a variety of p-value thresholds, could be used in PRS-by-environment interaction studies. Using the optimal PRS is an analytical choice akin to only examining variables with main effects in any interaction analysis.

An alternative BMI PRS (specifically, one optimised to predict SES) was significantly associated with SES. This demonstrates both that SES can be predicted from genetic data, and that there is an overlap of the genetic influences on BMI and SES. The analyses in this study used the PRS optimised for BMI (which was not significantly associated with SES) as this best captures the overall influence of the genome on BMI. The modelled interaction term then examines how this genomic effect alters in the presence of the social environment. It would be possible to use the PRS optimised for SES instead. This would focus on the genetic overlap between the two traits; however, the interaction term would then examine how this shared genetic component altered in the presence of the environment with which it is associated. It is unclear what the implications of a significant interaction would be in this case.

A central issue of gene-environment interaction studies is the definition of the environment^28^. Although the measures of parenting and SES used in this analysis have previously been used successfully to capture their respective constructs, they both differ from possible alternatives^41,42^. Previous research on the effect of parenting style on BMI has examined parental control and involvement. The concepts of parental punitive discipline and parental warmth used in this analysis are similar. However, parental control reflects aspects of both constructive and punitive discipline, whereas the discipline measure used in this analysis focusses on punitive discipline alone. As such, the parenting style measure used in this analysis differs from that used elsewhere in the BMI literature. Furthermore, parenting behaviour is highly complex and multi-faceted, and the measure of parental style used herein can only approximate the overall effect of parenting. In part, the secondary analyses performed using SES as the environment of interest reflects the need to examine the broader effects of social environment. However, this measure is also only one means of capturing a complex construct, and different measures of the social environment could yield different results.

Results from the study of change across adolescence need careful interpretation. The random intercepts used in the construction of the change phenotype are highly correlated with BMI at age 11 (r ≈ 0.9). As such, the inclusion of BMI at 11 as a covariate in the analysis of BMI change largely accounts for influences on pre-adolescent BMI. The non-significant association of social environment with this phenotype may thus reflect the continuation of effects from pre-adolescence, rather than an absence of effect during adolescence.

The change in BMI across adolescence was modelled as a linear slope for each participant. Previous analyses have suggested BMI change across adolescence may follow a quadratic slope, and so a linear model may not best explain the change in these data^43^. However, the limited number of assessments of BMI available prevent clear conclusion of the appropriate model for these data.

Finally, the definition of BMI in this study used weight and height as measured by the participants or their families, rather than direct measurement by researchers. Specifically, height and weight were ascertained in this cohort via self-report from the participants, as part of a larger questionnaire booklet^31^. Studies comparing self-reported to objectively-measured BMI report a general trend for height to be overestimated and weight to be underestimated, which consequently results in underestimates of BMI^38^. Discrepancies tend to be greater in females, and increase with weight and age^38^. Although this discrepancy has largely been observed in adults, there is also similar evidence reported in children^39,40^. Therefore, although reported discrepancies tend to be small, and participants were excluded if they reported impossible or inconsistent heights or weights, this reliance on self-report may result in measurement error and inaccuracies in the definition of BMI. Due to the breadth of phenotype collection in TEDS, it is not practical for all phenotypes to be collected via objective measurement.

### Interpretation

Genetic risk, modelled as a PRS derived from a cohort mostly comprising adult participants, captures a significant amount of variance both in BMI pre-adolescence, and in change in BMI across adolescence. This suggests that the genetic effects on BMI are (at least partly) stable across the lifespan. This is consistent with findings from quantitative genetic studies, which suggest a sizable component of genetic influence on BMI remains from childhood into adulthood, and with high genetic correlations (r_g_ = 0.73) reported in a meta-analysis of GWAS studies in children^7,33,44^. It also corroborates similar findings from previous analyses that used risk scores constructed only from significantly associated variants^10–12^.

Stratified analyses did not suggest a sex-specific effect in this study. Although genetic risk was significantly associated with change in BMI across adolescence in females only, the absence of a significant genetic risk-by-sex interaction in the main analysis suggests this could result from measurement error alone. However, the demographic differences between females and males observed in the cohort argue that stratifying analyses by sex is appropriate in studying influences (genetic and otherwise) on BMI at this age.

The effect of parental warmth and discipline in this study was of nominal significance, and did not pass correction for multiple testing. However, when SES is not included in the model, the effect is larger (and would have passed correction for multiple testing had secondary analyses with SES not been performed). The analyses presented have reasonable power. Cohen suggested f^2^ = 0.02 as a small effect, and all post-hoc power calculations show all analyses within this paper had lower f^2^ than this^35^. As such, while we cannot exclude an effect of parental warmth and punitive discipline on BMI, these results suggest any such effect is likely to be very small.

In contrast to the effect of parenting in this analysis, parental SES was associated with BMI at 11, suggesting an effect of the social environment from sources other than parenting style alone. Furthermore, the interaction between genetic influences and SES reached a nominal level of significance in the analysis of BMI change across adolescence, and it may be of interest to explore this interaction in a larger cohort. However, conclusions from the analysis using SES must be tempered by the fact that these are secondary analyses, related to the initial hypothesis (that BMI and parenting act together to influence BMI) but not explicitly specified.

The components of variance captured by parenting and SES are correlated, and including one in the model diminishes the effect of the other. The social environment is a complex construct that is likely to reflect and to be influenced by many factors in the wider environment. As such, further investigation to identify the precise component of the social environment that influences juvenile BMI would be of value.

The increasing rate of obesity is a developing public health crisis, and BMI, although imperfect, is a useful proxy of overall metabolic health^45^. An improved understanding of the factors affecting BMI in late childhood and adolescence could provide useful information in addressing this crisis. It is likely that the majority of obesity does not stem from single factor causes (such as mutations in the leptin system), but rather from the upper extreme of the normal population distribution of BMI^46^. TEDS is a population cohort, and is not enriched for juvenile obesity. However, studying this cohort can yield insight about the aetiology of BMI within the normal distribution, which may, in turn, be informative about the extremes of that distribution.

The generalisability of genetic findings from a population cohort to the genetics of obesity relies on the assumption that the genetic factors that predispose individuals to extreme BMI influence variance across the BMI spectrum. Yet it may be that distinct genetic influences predispose to extremely low and extremely high BMI^47^. Other anthropometric traits show evidence for distinct contributions of (often rare or non-additive) genetic effects at the extremes. For example, extremely short individuals show less depletion of a polygenic risk score for height than would be expected from their position in the overall spectrum, indicating a stronger influence of rare or non-additive effects (genetic or otherwise) at this extreme^48^. However, a reanalysis of the GIANT BMI GWAS, comparing genetic influences in the tails of the distribution with those in the distribution as a whole reported no systematic differences in the additive effects of common variants (although this does not preclude rare variant effects or effects acting in a non-additive manner^47^). This mirrors similar findings in young adults^49^. Furthermore, the most recent genomic study of anorexia nervosa (which is characterised in part by extremely low BMI) identified negative genetic correlations with extremely high BMI (rg = −0.29) and with BMI in the normal range (rg = −0.25^50^). Together, these data provide tentative evidence supporting a role for a shared, common, additive genetic effect across the range of BMI (although genetic correlations are not necessarily transitive^51^).

This study has shown a stable effect of genetic variants (from a meta-analysis predominantly of adult genome-wide association studies of BMI) capturing variance in BMI in children entering adolescence, and also capturing variance in the trajectory of BMI growth across adolescence. SES is associated with BMI pre-adolescence, but parenting style has at most a small effect. The availability of powerful genome-wide meta-analyses and the decreasing cost of obtaining genome-wide genotype data have increased the potential for performing genome-by-environment interaction studies to identify influential factors underlying important phenotypes in public health.

## Materials and Methods

### Analysis sample

Data on BMI at 11 years old, child perceptions of parental warmth and punitive discipline, covariates of interest (including parental socioeconomic status) and genome-wide genotype data were available from 3414 unrelated participants from TEDS. A subset of the cohort (N = 1943) also had BMI data at a later assessment (14 years old, 16 years old, or both). Although TEDS is a twin cohort, only one member of each twin pair was genotyped, and so no twin pairs are included in this analysis. The sample was restricted to individuals self-identifying as White Western European, and ancestry outliers were removed based on genotype data (Supplementary Material^52^).

### Phenotype definition

BMI was calculated from self-reported height and weight, following exclusion of participants reporting impossible values for these measurements or who reported a reduction of >2 cm in height from earlier reports (to allow for measurement error). BMI was transformed using a natural logarithm to address positive skew in the distribution.

Parenting was defined as the combined results from the child-report sections of the shortened Parental Feelings Questionnaire (PFQ) and the Parental Strategies Questionnaire (PSQ), which measure parental warmth and quality of parental discipline respectively^53,54^. The PFQ consists of seven statements designed to assess the warmth of the parent-child relationship (for example, “I feel close to my Mum/Dad”, answered *very true/quite true/not true*). Similarly, the PSQ contains four three-point scales assessing parental actions when the child misbehaved, such as “When I misbehave I am told off or shouted at”, answered *not true/quite true/very true*. Both scales were scored such that higher scores reflected less parental warmth and more punitive discipline respectively. Total scores were standardised and summed to give an overall parenting style variable^41^.

Covariates were included to control for the effects of age (in days) at assessment, sex, pubertal development and SES at birth, as well as eight principal components from genome-wide genotype data (to control for population stratification), and a binary variable to capture differences between genotyping waves. Pubertal development was assessed using the Petersen Pubertal Development Scale (PDS), which has five items assessing the progress of markers of puberty^55^. This includes three general questions (for example “*would you say your growth-spurt has not yet begun/barely begun/definitely begun/completed”*) and two sex-specific questions (assessing breast development and menstruation in females, and hair growth and voice deepening in males). The overall score is a mean average of these five items.

A composite measure of SES was derived at the birth of the participants based on measures of maternal and paternal qualifications and occupations, and maternal age at first childbirth, which were standardised and summed^42^. Specifically, maternal and paternal qualifications at birth were scored from 1 (no qualifications) to 8 (postgraduate qualifications), and occupations were scored from 1 (unskilled) to 9 (managerial). Maternal age at first childbirth was encoded in years. Higher composite scores reflect higher SES.

### Genotype data

Genome-wide genotyping data was obtained in two waves of genotyping, and imputed using minimac3 to the Haplotype Reference Consortium reference data (Supplementary Material^52,56–58^). Details on quality control and imputation are included in the Supplementary Methods. Following QC, genotyped or imputed data from 5147884 variants was available on 6710 participants, of which 3414 had the appropriate phenotypic data to be included in analysis.

### Polygenic risk scoring

Polygenic risk scores (PRS) were generated in the TEDS cohort using the results from the largest published meta-analysis of BMI genome-wide association studies^6^. The risk score capturing the most variance in BMI at 11 years old was obtained using the default settings in PRSice, which identifies the most predictive score by high-resolution polygenic risk scoring^59^. Specifically, the risk allele of genetic variants present in both the GIANT 2015 BMI GWAS and the cohort under study were weighted by their reported effect sizes from the GIANT study^6^. Ten thousand risk scores were generated stepwise by including variants with p-values less than a progressively more liberal threshold (0.0001 to 0.5, increasing by 0.00005 each step). Risk scores were regressed on BMI at 11 years old, including as covariates eight principal components to control for population stratification, and a binary variable to capture differences between genotyping waves. The risk score explaining the most variance in BMI at 11 years old was taken forward for analyses.

### Statistical analysis

Linear models were constructed in R to test the individual and interactive effects of parenting and genetic risk on BMI at 11. Covariates were included to control for the effects of age (in days) at assessment, sex, pubertal development and SES at birth, as well as eight principal components from genome-wide genotype data (to control for population stratification), and a binary variable to capture differences between genotyping waves. Continuous variables and covariates (that is, all except sex and genotyping wave) were standardised to produce standardised betas. Pairwise correlations between variables and covariates were calculated to assess the impact of multicollinearity. When interactions between parenting and genetic risk were included in the linear model, all covariate-by-parenting and covariate-by-genetic risk interactions were also included^60^.

A subset of the cohort (N = 1943) had BMI data at a later assessment (14 years old, 16 years old, or both). 154 individuals with BMI data at 16 had no age information recorded, so their age at 16 was imputed from age at 11 (Supplementary Material). BMI was regressed on time from initial assessment in random effects models using the *lme4* package in R^61^. The resulting slope for each individual was used as the phenotype in further linear models to determine the effects of genetic risk and parenting at 11 years old on change in BMI across adolescence, controlling for covariates as in the previous analysis.

Stratified secondary analyses were performed to assess sex-specific effects on BMI at 11 years and on change in BMI across adolescence. Post-hoc power calculations were performed using the *pwr* package in R to assess the strength of evidence provided by this study. Specifically, the minimum f^2^ values that the analyses had 80% and 90% power to detect were calculated, and the power of the analysis to detect observed f^2^ values for social environmental variables and genetic risk were calculated^35^. Cohen’s f^2^ is a measure of effect size suitable for assessing the contribution of a single variable in a multiple regression^35^.

During analysis with parental warmth and discipline, it became clear that SES competed with parenting to explain variance in BMI at 11. When SES was included as a covariate in the model, the proportion of variance explained by parenting was diminished compared to when SES was not included. Analyses were thus repeated with SES as the environmental variable of interest (and parenting as a covariate).

In total, twelve analyses were performed, with three basic models (full model, female-only and male-only) for two phenotypes (BMI at 11 and change in BMI across adolescence) with two environments of interest (parenting and SES).

### Sensitivity analyses

The analytical decisions made in the course of statistical analysis may affect the outcome. Accordingly a number of sensitivity analyses were performed. Alterations to the scale of variables affects the interpretation of interaction models. As such, all reported analyses were rerun without transforming BMI (Supplementary Tables S6–9).

Alternative meta-analyses were considered as the source dataset for generating polygenic risk scores. These were the European subset of the GIANT 2015 meta-analysis, and the meta-analysis of a smaller cohort of children^6,7^. The cross-ethnic meta-analysis was selected for the main analysis as it was largest and expected to provide the most power. Sensitivity analyses were performed using the alternative sources (Supplementary Note).

Much of the literature on the effect of gene-environment interactions on BMI has examined variation in the FTO gene, particularly the variant rs9939609^25–27,62^. To allow comparison with the literature, analyses were repeated with rs9939609 in place of the PRS (Supplementary Material).

The risk score capturing the most variance in BMI at 11 years old was used in all analyses because it acts as the strongest proxy for the effect of the genome on BMI at 11 in this cohort. However, this score is likely to be overfit to this particular cohort. Sensitivity analyses were performed with the full set of variants present in both GIANT and this cohort (that is, a risk score selected with a p-value threshold of 1) to test the importance of this unknown overfit (Supplementary Tables S10 and S11). This risk score will contain all of the signal possible to be captured using the polygenic risk scoring method, but will also contain a large amount of stochastic noise.

Parental style assessments are inherently subjective, and as such each twin in a pair may differently report parental actions. Co-twin report of parental style was substituted into the main analyses to test the effect of this potential difference.

Parental style may change with the age of parents. Maternal and paternal age was added as an additional covariate to test whether this altered the conclusions of the main analyses.

### Ethics

Parents provided informed consent for each part of the study prior to data collection. King’s College London’s Ethics Committee provided ethical approval. The research was performed in compliance with the Declaration of Helsinki.

### Data availability statement

The Twins Early Development Study dataset is a controlled-access dataset that is accessible through collaboration with core TEDS researchers. A full explanation of the data access policy can be found at http://www.teds.ac.uk/research/collaborators-and-data/teds-data-access-policy.

## Electronic supplementary material

Supplementary Material
