# Supplementary material for: Individual and shared effects of social environment and polygenic risk scores on adolescent body mass index
Source: Sci Rep. 2018 Apr 20;8:6344. doi: 10.1038/s41598-018-24774-5 (PMC5910407; doi:10.1038/s41598-018-24774-5)
Supplement: Supplementary file 1 — Supplementary Material [file 41598_2018_24774_MOESM1_ESM.doc]

**Supplementary Material for *"* Individual and shared effects of social environment and polygenic risk scores on adolescent body mass index "**

Jonathan R.I. Coleman, Eva Krapohl, Thalia C. Eley, Gerome Breen

**Supplementary Methods**

Genotyping protocol and quality control

Genome-wide genotype data was obtained in two waves of collection. In the first wave, DNA from 3,665 samples was extracted from buccal cheek swabs and genotyped at Affymetrix, Santa Clara, California, USA. Samples were successfully hybridized to AffymetrixGeneChip 6.0 SNP genotyping arrays (<http://www.affymetrix.com/support/technical/datasheets/genomewide_snp6_datasheet.pdf>) using experimental protocols recommended by the manufacturer (Affymetrix Inc., Santa Clara, CA). The raw image data from the arrays were normalized and pre-processed at the Wellcome Trust Sanger Institute, Hinxton, UK for genotyping as part of the Wellcome Trust Case Control Consortium 2 (<https://www.wtccc.org.uk/ccc2/>) according to the manufacturer’s guidelines (<http://www.affymetrix.com/support/downloads/manuals/genomewidesnp6_manual.pdf>). Genotypes were called using CHIAMO (<https://mathgen.stats.ox.ac.uk/genetics_software/chiamo/chiamo.html>).

In the second wave, DNA for 4,649 individuals was extracted from saliva samples and hybridized to HumanOmniExpressExome-8v1.2 genotyping arrays at the Institute of Psychiatry, Psychology and Neuroscience Genomics & Biomarker Core Facility. The raw image data from the array were normalised, pre-processed, and filtered in GenomeStudio following internal protocols (<http://confluence.brc.iop.kcl.ac.uk:8090/display/PUB/Production+Version%3A+Illumina+Exome+Chip+SOP+v1.4>). Prior to genotype calling 869 multi-mapping SNPs and 353 samples with call rate <.95 were removed. ZCall was used to augment the genotype calling for samples and SNPs that passed the initial QC [^1^](#_ENREF_1).

After initial quality control and genotype calling, the same quality control was separately performed on samples from both waves using PLINK, R and vcftools [^2-5^](#_ENREF_2).

Samples were removed from subsequent analyses on the basis of call rate (<0.99), suspected non-European ancestry, heterozygosity, array signal intensity, and relatedness. SNPs were excluded if the minor allele frequency was <.05%, if more than 1% of genotype data were missing, or if the Hardy Weinberg p-value was lower than 10-5. Non-autosomal markers and insertion-deletions were removed. Association between the SNP and the array, batch, or plate on which samples were genotyped was calculated; SNPs with an effect p-value < 10^-3^ were excluded. A total sample of 6,710 samples, with 3,617 individuals and 600,034 SNPs genotyped on Illumina and 3,093 individuals and 525,859 SNPs genotyped on Affymetrix remained after quality control.

Genotypes from the two arrays were separately imputed using the Haplotype Reference Consortium [^6^](#_ENREF_6) and Minimac3 1.0.13 available on the Michigan Imputation Server as reference data. A series of quality checks was performed before data from the two waves were merged (e.g. array effects, allele frequencies by imputation quality). For the present analyses we limited our analyses to variants genotyped or imputed at info >.95 on both arrays, allele frequency difference between arrays smaller than 5%, and Hardy Weinberg p-value was greater than 10^-5^. Using these criteria, 5,147,884 genotyped and well-imputed SNPs were retained for the analyses.

Principal component analysis was performed on a subset of 42,859 common (MAF>5%) autosomal SNPs found on the HapMap3 data [^9^](#_ENREF_9), after stringent pruning to remove markers in high linkage disequilibrium (r2 > 0.1) and excluding high linkage disequilibrium genomic regions so as to ensure that only genome-wide effects were detected.

Multiple imputation of BMI change data

154 individuals with BMI data at 16 had no age information recorded, so their age at 16 was imputed from age at 11 in a twenty-fold multiple imputation use the *mi* package in R [^10-12^](#_ENREF_10). Results from each multiple imputation were used to construct a random effects model (random intercepts and random slopes) of BMI regressed on time from initial assessment, using the *lme4* package in R [^13^](#_ENREF_13). The random coefficient associated with time for each individual was averaged across the twenty models. The average coefficient was then used as the phenotype in further linear models to determine the effects of genetic risk and parenting at 11 years old on change in BMI across adolescence.

**Supplementary Notes**

Sensitivity analyses using alternative PRS

The main analyses used a PRS derived from the all-ancestries analysis of the GIANT adult BMI GWAS meta-analysis [^14^](#_ENREF_14). There are good justifications to consider the European-only analyses from the adult GWAS (as the TEDS sample is predominantly of White Western European ancestry) and a recent child BMI GWAS meta-analysis as alternative base GWAS for generating PRS [^14-16^](#_ENREF_14). Analyses were repeated using these PRS. Conclusions from the replication with the European subset PRS differed in that the main effect of socioeconomic status on change in BMI across adolescence in males was not significant when PRS was in the model (*p* = 0.00451). Using the child BMI PRS from Felix et al (2016) did not alter the conclusions from the main analyses.

Interaction analyses with FTO rs9939609

To enable comparison to this previous literature, analyses were re-run using the number of A alleles of rs9939609 in place of the polygenic risk score. Results were similar to those obtained with the full polygenic risk score, but showed smaller effects (Supplementary Tables 4 and 5).

Effect of transforming BMI

Alterations to the scale of variables affects the interpretation of interaction models. As such, all reported analyses were rerun without transforming BMI (Supplementary Tables 6-9). Although the exact results differed between this and the main analysis, the conclusions drawn remained the same.

Using a polygenic risk score including all variants

The risk score capturing the most variance in BMI at 11 years old was used in all analyses because it acts as the strongest proxy for the effect of the genome on BMI at 11 in this cohort. However, this score is likely to be overfit to this particular cohort. Sensitivity analyses were performed with the full set of variants present in both GIANT and this cohort (that is, a risk score selected with a p-value threshold of 1) to test the importance of this unknown overfit (Supplementary Tables 10 and 11). Although the exact results differed between this and the main analysis, the conclusions drawn remained the same.

Co-twin assessment of parental style

Substituting co-twin assessments for focal twin assessments in analyses of BMI at 11 and change in BMI across adolescence did not alter results (that is, the conclusions drawn remained the same).

Inclusion of maternal and paternal age as covariates

Including maternal and paternal age as covariates in analyses of BMI at 11 and change in BMI across adolescence did not alter results (that is, the conclusions drawn remained the same).

**Supplementary References**

1 Goldstein, J. I. *et al.* zCall: a rare variant caller for array-based genotyping: genetics and population analysis. *Bioinformatics (Oxford, England)* **28**, 2543-2545, doi:10.1093/bioinformatics/bts479 (2012).

2 Purcell, S. *et al.* PLINK: a tool set for whole-genome association and population-based linkage analyses. *American journal of human genetics* **81**, 559-575, doi:10.1086/519795 (2007).

3 Chang, C. *et al.* Second-generation PLINK: rising to the challenge of larger and richer datasets. *GigaScience* **4**, 7 (2015).

4 Team, R. C. (2014).

5 Danecek, P. *et al.* The variant call format and VCFtools. *Bioinformatics* **27**, 2156-2158, doi:10.1093/bioinformatics/btr330 (2011).

6 McCarthy, S. *et al.* A reference panel of 64,976 haplotypes for genotype imputation. *Nature genetics* **48**, 1279-1283, doi:10.1038/ng.3643 (2016).

7 Howie, B., Fuchsberger, C., Stephens, M., Marchini, J. & Abecasis, G. R. Fast and accurate genotype imputation in genome-wide association studies through pre-phasing. *Nature genetics* **44**, 955-959, doi:10.1038/ng.2354 (2012).

8 Fuchsberger, C., Abecasis, G. R. & Hinds, D. A. minimac2: faster genotype imputation. *Bioinformatics* **31**, 782-784 (2015).

9 Consortium, I. H. Integrating common and rare genetic variation in diverse human populations. *Nature* **467**, 52-58 (2010).

10 Rubin, D. B. *Multiple imputation for nonresponse in surveys*. Vol. 81 (John Wiley & Sons, 2004).

11 Graham, J. W., Olchowski, A. E. & Gilreath, T. D. How many imputations are really needed? Some practical clarifications of multiple imputation theory. *Prevention Science* **8**, 206-213 (2007).

12 Su, Y. S., Gelman, A., Hill, J. & Yajima, M. Multiple Imputation with Diagnostics (mi) in R: Opening Windows into the Black Box. *Journal of Statistical Software* **45**, 1-31 (2011).

13 Bates, D., Mächler, M., Bolker, B. & Walker, S. Fitting linear mixed-effects models using lme4. *arXiv preprint arXiv:1406.5823* (2014).

14 Locke, A. E. *et al.* Genetic studies of body mass index yield new insights for obesity biology. *Nature* **518**, 197-206, doi:10.1038/nature14177 (2015).

15 Trzaskowski, M. *et al.* First genome-wide association study on anxiety-related behaviours in childhood. *PLoS ONE* **8**, e58676, doi:10.1371/journal.pone.0058676 (2013).

16 Felix, J. F. *et al.* Genome-wide association analysis identifies three new susceptibility loci for childhood body mass index. *Hum Mol Genet* **25**, 389-403, doi:10.1093/hmg/ddv472 (2016).

17 Keller, M. C. Gene × Environment Interaction Studies Have Not Properly Controlled for Potential Confounders: The Problem and the (Simple) Solution. *Biological psychiatry* **75**, 18-24 (2014).

Supplementary Tables

|  | log(BMI) | BMI Change | BMI PRS | Parenting | SES | Sex | Age | Puberty | Wave | PC1 | PC2 | PC3 | PC4 | PC5 | PC6 | PC7 | PC8 |
| --- | --- | --- | --- | --- | --- | --- | --- | --- | --- | --- | --- | --- | --- | --- | --- | --- | --- |
| log(BMI) | - | **3.3x10^-135^** | **1.9x10^-37^** | **0.026** | **4.0 x10^-8^** | **0.0029** | **2.3x10^-18^** | **2.7x10^-44^** | 0.93 | 0.60 | 0.90 | 0.63 | 0.36 | 0.46 | 0.44 | **0.035** | 0.88 |
| BMI Change | **-0.52** | - | 0.26 | 0.054 | **0.0016** | 0.77 | **0.0089** | **1.9x10^-5^** | 0.22 | **0.024** | 0.56 | 0.83 | **0.0051** | 0.79 | 0.63 | **0.042** | 0.80 |
| BMI PRS | **0.22** | -0.026 | - | 0.43 | 0.13 | 0.54 | 0.35 | 0.078 | 0.81 | **0.037** | 0.80 | 0.45 | 0.77 | 0.77 | 0.81 | 0.80 | 0.48 |
| Parenting | **0.038** | -0.044 | 0.013 | - | **3.7x10^-8^** | **9.7x10^-8^** | **0.0025** | 0.63 | 0.97 | 0.91 | 0.61 | 0.93 | 0.71 | 0.14 | 0.45 | 0.37 | 0.86 |
| SES | **-0.094** | **0.072** | -0.026 | **-0.094** | - | 0.86 | **1.8x10^-5^** | **2.3x10^-4^** | 0.14 | 0.18 | 0.19 | **1.4 x10^-5^** | 0.91 | 0.42 | 0.10 | 0.78 | 0.33 |
| Sex | **-0.051** | -0.0067 | 0.010 | **0.091** | 0.0030 | - | 0.55 | **9.2x10^-72^** | 0.12 | 0.071 | 0.32 | 0.24 | 0.98 | 0.75 | 0.25 | 0.13 | 0.76 |
| Age | **0.15** | **-0.059** | -0.016 | **-0.052** | **-0.073** | -0.01 | - | **3.7x10^-77^** | 0.31 | 0.14 | 0.60 | 0.58 | 0.28 | 0.92 | 0.77 | 0.86 | 0.32 |
| Puberty | **0.24** | **-0.097** | 0.030 | -0.0083 | **-0.063** | **-0.30** | **0.31** | - | 0.57 | 0.58 | 0.88 | 0.25 | 0.47 | 0.50 | 0.43 | 0.45 | 0.98 |
| Wave1 | -0.0016 | 0.028 | -0.0042 | -6.0 x10^-4^ | 0.025 | -0.027 | -0.017 | -0.0099 | - | **< 10^-150^** | **5.1x10^-92^** | **0.00032** | 0.22 | 0.95 | 0.10 | 0.31 | 0.28 |
| PC1 | -0.0090 | **0.051** | **-0.036** | 0.0021 | 0.023 | -0.031 | 0.025 | 0.0096 | **0.72** | - | 0.79 | 0.18 | 0.56 | 0.26 | 0.22 | 0.79 | 0.23 |
| PC2 | 0.0022 | -0.013 | 0.0044 | 0.0088 | -0.022 | -0.017 | 0.0091 | -0.0027 | **0.34** | -0.0045 | - | 0.63 | 0.70 | 0.40 | 0.29 | 0.28 | 0.12 |
| PC3 | 0.0083 | 0.0049 | -0.013 | -0.0016 | **0.074** | 0.020 | -0.0096 | -0.020 | **0.061** | -0.023 | 0.0081 | - | 0.19 | 0.42 | 0.10 | 0.70 | 0.68 |
| PC4 | 0.016 | **-0.064** | 0.0050 | -0.0064 | -0.0019 | -4.6x10^-4^ | -0.018 | -0.012 | -0.021 | -0.0099 | -0.0066 | 0.023 | - | 0.25 | 0.86 | 0.92 | 0.87 |
| PC5 | -0.013 | 0.0059 | 0.0051 | 0.025 | -0.014 | -0.0054 | 0.0018 | -0.012 | 0.001 | 0.019 | -0.015 | -0.014 | -0.020 | - | 0.94 | 0.68 | 0.60 |
| PC6 | 0.013 | 0.011 | -0.0041 | -0.013 | 0.028 | 0.020 | 0.0051 | -0.014 | -0.028 | -0.021 | -0.018 | 0.028 | -0.0031 | 0.0012 | - | 0.84 | 0.28 |
| PC7 | **-0.036** | **-0.046** | 0.0044 | 0.015 | 0.0047 | 0.026 | -0.0030 | -0.013 | -0.017 | -0.0046 | -0.018 | -0.0066 | -0.0018 | 0.0071 | -0.0034 | - | 0.13 |
| PC8 | 0.0026 | 0.0058 | -0.012 | 0.0030 | 0.017 | -0.0052 | -0.017 | -4.0x10^-4^ | 0.018 | 0.021 | -0.026 | -0.0072 | 0.0030 | -0.0089 | 0.019 | 0.026 | - |

Supplementary Table S1

Supplementary Table S1: Correlations between phenotypes and variables in the analyses (Pearson pairwise product-moment correlation; lower triangle) and associated *p*-values (upper triangle). Correlations with BMI Change are in N=1943, all other correlations N=3414.
Nominally significant correlations are marked in bold (*p* < 0.05).

Supplementary Table S2a

| **BMI at 11 years old, with parenting** | | | | | | | | | | |
| --- | --- | --- | --- | --- | --- | --- | --- | --- | --- | --- |
|  | | **Full cohort (N = 3414)** | | | **Females (N = 1750)** | | | **Males (N = 1664)** | | |
| **Coefficient** | | **B** | **SE** | ***p*** | **B** | **SE** | ***p*** | **B** | **SE** | ***p*** |
| Null Model | Sex  **Age SES**  **Pubertal development**  Wave  PC1  PC2  PC3  PC4  PC5  PC6  PC7  PC8 | 0.0260  **0.0795**  **-0.0766**  **0.211**  0.0598  -0.0312  -0.00982  0.0149  0.0193  -0.0101  0.0169  -0.0329  0.00575 | 0.0349  **0.0176**  **0.0167**  **0.0183**  0.0566  0.0260  0.0191  0.0167  0.0166  0.0166  0.0166  0.0166  0.0166 | 0.457  **6.12x10^-6^**  **4.42x10^-6^**  **5.44x10^-30^**  0.291  0.230  0.607  0.372  0.245  0.541  0.307  0.0472  0.728 | -  0.0346  -0.0740  0.276  0.151  -0.0443  -0.0429  0.0190  0.00107  -0.00566  0.0246  -0.0190  0.0181 | -  0.0248  0.0230  0.0247  0.0780  0.0353  0.0262  0.0231  0.0229  0.0229  0.0229  0.0229  0.0229 | -  0.162  **0.00131**  **4.38x10^-28^**  0.0532  0.211  0.102  0.410  0.963  0.805  0.281  0.407  0.431 | -  **0.126**  **-0.0796**  **0.106**  -0.0566  -0.0107  0.0267  0.00822  0.0402  -0.0148  0.00705  -0.0461  -0.0155 | -  **0.0249**  **0.0243**  **0.0249**  0.0826  0.0386  0.0280  0.0244  0.0241  0.0241  0.0241  0.0240  0.0241 | -  **4.90x10^-7^**  **0.00106**  **2.30x10^-5^**  0.493  0.782  0.340  0.736  0.0954  0.538  0.770  0.0553  0.520 |
| Null model + parental style | | 0.0378 | 0.0167 | 0.0239 | 0.0413 | 0.0230 | 0.0730 | 0.0364 | 0.0242 | 0.133 |
| Null model **+ BMI PRS** | | **0.210** | **0.0162** | **1.59x10^-37^** | **0.192** | **0.0224** | **3.04x10^-17^** | **0.231** | **0.0234** | **2.68x10^-22^** |
| Null model + Parental style **+ BMI PRS** | | 0.0360  **0.210** | 0.0163  **0.0162** | 0.0273  **1.83x10^-37^** | 0.0407  **0.191** | 0.0225  **0.0224** | 0.0712  **3.03x10^-17^** | 0.0330  **0.230** | 0.0235  **0.0234** | 0.160  **3.19x10^-22^** |
| Null model + Parental style x BMI PRS | | 0.00642 | 0.0172 | 0.709 | -0.00627 | 0.0240 | 0.794 | 0.0296 | 0.0252 | 0.241 |

Supplementary Table S2a: Effects of adding variables and interactions to the null model (uppermost line) predicting variance in BMI at 11 years old, with parenting as the environment of interest. Significant (p<0.00417) terms are in bold. Interactions include all main effects, covariates and covariate interaction terms [^17^](#_ENREF_17).

Supplementary Table S2b

| **BMI at 11 years old, with socioeconomic status** | | | | | | | | | | |
| --- | --- | --- | --- | --- | --- | --- | --- | --- | --- | --- |
|  | | **Full cohort (N = 3414)** | | | **Females (N = 1750)** | | | **Males (N = 1664)** | | |
| **Coefficient** | | **B** | **SE** | ***p*** | **B** | **SE** | ***p*** | **B** | **SE** | ***p*** |
| Null Model | Sex  **Age** Parenting  **Pubertal development**  Wave  PC1  PC2  PC3  PC4  PC5  PC6  PC7  PC8 | 0.0187  **0.0868**  0.0448  **0.212**  0.0581  -0.0329  -0.00838  0.00950  0.0199  -0.0102  0.0156  -0.0338  0.00455 | 0.0351  **0.0176**  0.0167  **0.0184**  0.0567  0.0261  0.0191  0.0167  0.0166  0.0166  0.0166  0.0166  0.0166 | 0.594  **8.39x10^-7^**  0.00726  **2.43x10^-30^**  0.306  0.207  0.661  0.57  0.229  0.538  0.349  0.0418  0.784 | -  0.0401  0.0490  **0.279**  0.142  -0.0421  -0.0384  0.0143  0.00277  -0.00473  0.0254  -0.0200  0.0174 | -  0.0248  0.0229  **0.0247**  0.0781  0.0354  0.0262  0.0231  0.0229  0.0229  0.0229  0.0229  0.0230 | -  0.107  0.0326  **1.22x10^-28^**  0.0687  0.235  0.143  0.536  0.904  0.837  0.268  0.382  0.449 | -  **0.135**  0.0426  **0.106**  -0.0507  -0.0170  0.0247  0.00178  0.0399  -0.0164  0.00354  -0.0466  -0.0173 | -  **0.0250**  0.0242  **0.0250**  0.0829  0.0387  0.0280  0.0243  0.0242  0.0241  0.0242  0.0241  0.0241 | -  **7.18x10^-8^**  0.0784  **2.46x10^-5^**  0.540  0.660  0.379  0.942  0.0991  0.497  0.884  0.0532  0.473 |
| Null model **+ SES** | | **-0.0730** | **0.0167** | **1.33x10^-5^** | **-0.0694** | **0.0231** | **0.00274** | **-0.0766** | **0.243** | **0.00167** |
| Null model **+ BMI PRS** | | **0.211** | **0.0162** | **7.95x10^-38^** | **0.192** | **0.0225** | **2.38x10^-17^** | **0.232** | **0.0235** | **1.75x10^-22^** |
| Null model **+ SES + BMI PRS** | | **-0.0682**  **0.210** | **0.0163**  **0.0162** | **3.11x10^-5^**  **1.83x10^-37^** | **-0.0663**  **0.191** | **0.0227**  **0.0224** | **0.00349**  **3.03x10^-17^** | **-0.0701**  **0.230** | **0.0237**  **0.0234** | **0.00309**  **3.19x10^-22^** |
| Null model + SES x BMI PRS | | -0.0336 | 0.0165 | 0.0413 | -0.0230 | 0.0233 | 0.324 | -0.0382 | 0.0239 | 0.111 |

Supplementary Table S2b: Effects of adding variables and interactions to the null model (uppermost line) predicting variance in BMI at 11 years old, with socioeconomic status as the environment of interest. Significant (p<0.00417) terms are in bold. Interactions include all main effects, covariates and covariate interaction terms [^17^](#_ENREF_17).

Supplementary Table S3a

| **BMI change across adolescence, with parenting** | | | | | | | | | | |
| --- | --- | --- | --- | --- | --- | --- | --- | --- | --- | --- |
|  | | **Full cohort (N = 1943)** | | | **Females (N = 1043)** | | | **Males (N = 900)** | | |
| **Coefficient** | | **B** | **SE** | ***p*** | **B** | **SE** | ***p*** | **B** | **SE** | ***p*** |
| Null Model | **BMI at 11**  Sex  Age SES  Pubertal development  Wave  PC1  PC2  PC3  **PC4**  PC5  PC6  **PC7**  PC8 | **-0.528**  -0.0281  0.0178  0.0113  0.0126  -0.0135  0.0353  -0.0157  0.0105  **-0.0588**  -0.00878  0.00908  **-0.0710**  0.00997 | **0.0201**  0.0410  0.0205  0.0196  0.0218  0.0665  0.0303  0.0224  0.0197  **0.0193**  0.0193  0.0193  **0.0194**  0.0193 | **1.56x10^-130^**  0.493  0.386  0.565  0.562  0.839  0.244  0.483  0.594  **0.00242**  0.650  0.639  **0.000251**  0.606 | **-0.546**  -  -0.0138  -0.0120  0.0206  0.0749  -0.00625  -0.00365  -0.00693  -0.0593  -0.0234  0.0134  -0.0666  0.00450 | **0.0275**  -  0.0286  0.0264  0.0293  0.0901  0.0404  0.0303  0.0266  0.0263  0.0263  0.0262  0.0263  0.0262 | **1.56x10^-74^**  -  0.628  0.650  0.482  0.406  0.877  0.904  0.794  0.0243  0.373  0.611  0.0114  0.864 | **-0.505**  -  0.0547  0.0407  0.0128  -0.130  0.0890  -0.0306  0.0290  -0.0536  0.00648  -0.00208  -0.0708  0.0207 | **0.0296**  -  0.0298  0.0294  0.0298  0.0992  0.0461  0.0335  0.0295  0.0290  0.0288  0.0290  0.0289  0.0289 | **1.55x10^-56^**  -  0.0673  0.167  0.669  0.190  0.0536  0.362  0.326  0.0650  0.822  0.943  0.0144  0.473 |
| Null model + parental style | | -0.00848 | 0.0196 | 0.666 | -0.0147 | 0.0265 | 0.580 | -9.79x10^‑5^ | 0.0291 | 0.997 |
| **Null model + BMI PRS** | | **0.0902** | **0.0197** | **4.96x10^-6^** | **0.105** | **0.0266** | **8.12x10^-5^** | 0.0738 | 0.0295 | 0.0124 |
| Null model + parental style  **+ BMI PRS** | | -0.00969  **0.0903** | 0.0195  **0.0197** | 0.620  **4.84x10^-6^** | -0.0159  **0.105** | 0.0263  **0.0266** | 0.546  **7.94x10^-5^** | -0.00134  0.0738 | 0.0290  0.0295 | 0.963  0.0125 |
| Null model + Parental style x BMI PRS | | 0.000551 | 0.0207 | 0.979 | 0.0209 | 0.0285 | 0.463 | -0.0416 | 0.0309 | 0.179 |

Supplementary Table S3a: Effects of adding variables and interactions to the null model (uppermost line) predicting change in BMI across adolescence, with parenting as the environment of interest. Significant (p<0.00417) terms are in bold. Interactions include all main effects, covariates and covariate interaction terms [^17^](#_ENREF_17).

Supplementary Table S3b

| **BMI change across adolescence, with socioeconomic status** | | | | | | | | | | |
| --- | --- | --- | --- | --- | --- | --- | --- | --- | --- | --- |
|  | | **Full cohort (N = 1943)** | | | **Females (N = 1043)** | | | **Males (N = 900)** | | |
| **Coefficient** | | **B** | **SE** | ***p*** | **B** | **SE** | ***P*** | **B** | **SE** | ***p*** |
| Null Model | **BMI at 11**  Sex  Age Parental style  Pubertal development  Wave  PC1  PC2  PC3  **PC4**  PC5  PC6  **PC7**  PC8 | **-0.528**  -0.0265  0.0162  -0.00936  0.0125  -0.0121  0.0351  -0.0161  0.0111  **-0.0587**  -0.00854  0.00938  **-0.0708**  0.00955 | **0.0200**  0.0412  0.0206  0.0196  0.0218  0.0664  0.0303  0.0224  0.0196  **0.0193**  0.0193  0.0193  **0.0194**  0.0193 | **2.99x10^-131^**  0.521  0.432  0.632  0.569  0.855  0.246  0.470  0.572  **0.00246**  0.659  0.628  **0.000262**  0.621 | **-0.544**  -  -0.0146  -0.0133  0.0206  0.0754  -0.00668  -0.00342  -0.00733  -0.0591  -0.0228  0.0128  -0.0670  0.00486 | **0.0275**  -  0.0286  0.0264  0.0293  0.0901  0.0404  0.0303  0.0265  0.0263  0.0263  0.0262  0.0263  0.0262 | **3.36x10^-74^**  -  0.611  0.613  0.484  0.403  0.869  0.910  0.783  0.0249  0.387  0.624  0.0109  0.853 | **-0.509**  -  0.0516  -0.00262  0.0104  -0.131  0.0915  -0.0307  0.0326  -0.0520  0.00784  -4.49x10^-5^  -0.0709  0.0198 | **0.0295**  -  0.0301  0.0291  0.0299  0.0993  0.0461  0.0336  0.0294  0.0290  0.0289  0.0290  0.0289  0.0289 | **1.11x10^-57^**  -  0.0862  0.928  0.727  0.189  0.0475  0.360  0.267  0.0738  0.786  0.999  0.0144  0.493 |
| Null model + SES | | 0.0106 | 0.0196 | 0.591 | -0.0135 | 0.0265 | 0.612 | 0.0407 | 0.0295 | 0.168 |
| **Null model + BMI PRS** | | **0.0904** | **0.0197** | **4.70x10^-6^** | **0.105** | **0.0266** | **8.19x10^-5^** | 0.0739 | 0.0295 | 0.0124 |
| Null model + SES  **+ BMI PRS** | | 0.00965  **0.0903** | 0.0195  **0.0197** | 0.622  **4.84x10^-6^** | -0.0152  **0.105** | 0.0264  **0.0266** | 0.565  **7.94x10^-5^** | 0.0405  0.0738 | 0.0294  0.0295 | 0.168  0.0125 |
| Null model + SES x BMI PRS | | -0.0494 | 0.0205 | 0.0159 | -0.0724 | 0.0282 | 0.0103 | -0.0152 | 0.0311 | 0.626 |

Supplementary Table S3b: Effects of adding variables and interactions to the null model (uppermost line) predicting change in BMI across adolescence, with socioeconomic status as the environment of interest. Significant (p<0.00417) terms are in bold. Interactions include all main effects, covariates and covariate interaction terms [^17^](#_ENREF_17).

Supplementary Table S4

| **Coefficient** | **B** | **SE** | ***p*** | **Adjusted R^2^** |
| --- | --- | --- | --- | --- |
| **BMI at 11 years old, with parenting** | | | | |
| Null model | Supplementary Table 2a | | | 0.0667 |
| (Null model) + Parental style | 0.0378 | 0.0167 | 0.0239 | 0.0678 |
| (Null model) **+ rs9939609** | **0.150** | **0.0239** | **4.64x10^-10^** | 0.0770 |
| (Null model) + Parental style  **+ rs9939609** | 0.0374  **0.149** | 0.0166  **0.0239** | 0.0247  **5.28x10^-10^** | 0.0781 |
| (Null model) + Parental style x rs9939609 | -0.0208 | 0.0245 | 0.395 | 0.0800 |
| **BMI at 11 years old, with SES** | | | | |
| Null model | Supplementary Table 2b | | | 0.0628 |
| Null model **+ SES** | **-0.0729** | **0.0167** | **1.33x10^-5^** | 0.0678 |
| Null model **+ rs9939609** | **0.149** | **0.0240** | **6.08x10^-10^** | 0.0734 |
| Null model **+** **SES** **+ rs9939609** | **-0.0714**  **0.149** | **0.0167**  **0.0239** | **1.89x10^-5^**  **5.28x10^-10^** | 0.0781 |
| Null model + SES x rs9939609 | -0.0237 | 0.0242 | 0.328 | 0.0794 |

Supplementary Table S4: Effects of adding variables and interactions to the null model predicting variance in BMI at 11 years old, with parenting as the environment of interest and FTO variant rs9939609 as the genotype of interest. Significant (p<0.00417) terms are in bold. Interactions include all main effects, covariates and covariate interaction terms [^17^](#_ENREF_17).

Supplementary Table S5

| **Coefficient** | **B** | **SE** | ***p*** | **Adjusted R^2^** |
| --- | --- | --- | --- | --- |
| **BMI change, with parenting** | | | | |
| Null model | Supplementary Table 3a | | | 0.277 |
| (Null model) + Parental style | -0.00848 | 0.0196 | 0.666 | 0.277 |
| (Null model) + rs9939609 | 0.0450 | 0.0285 | 0.114 | 0.277 |
| (Null model) + Parental style  + rs9939609 | -0.00702  0.0452 | 0.0196  0.0285 | 0.720  0.112 | 0.277 |
| (Null model) + Parental style x rs9939609 | 0.00651 | 0.0289 | 0.821 | 0.274 |
| **BMI change, with SES** | | | | |
| Null model | Supplementary Table 3b | | | 0.277 |
| Null model + SES | 0.0106 | 0.0196 | 0.591 | 0.277 |
| Null model + rs9939609 | 0.0454 | 0.0285 | 0.111 | 0.277 |
| Null model + SES + rs9939609 | 0.0121  0.0452 | 0.0196  0.0285 | 0.616  0.112 | 0.277 |
| Null model + SES x rs9939609 | 0.0180 | 0.0293 | 0.541 | 0.279 |

Supplementary Table S5: Effects of adding variables and interactions to the null model predicting variance in BMI at 11 years old, with SES as the environment of interest and FTO variant rs9939609 as the genotype of interest. Significant (p<0.00417) terms are in bold. Interactions include all main effects, covariates and covariate interaction terms [^17^](#_ENREF_17).

Supplementary Table S6a

| **Untransformed BMI at 11 years old, with parenting** | | | | | | | | | | |
| --- | --- | --- | --- | --- | --- | --- | --- | --- | --- | --- |
|  | | **Full cohort (N = 3414)** | | | **Females (N = 1750)** | | | **Males (N = 1664)** | | |
| **Coefficient** | | **B** | **SE** | ***p*** | **B** | **SE** | ***p*** | **B** | **SE** | ***p*** |
| Null Model | Sex  **Age SES**  **Pubertal development**  Wave  PC1  PC2  PC3  PC4  PC5  PC6  PC7  PC8 | 0.0165  **0.0750**  **-0.0831**  **0.203**  0.0507  -0.0283  -0.0152  0.0159  0.0209  -0.0106  0.0200  -0.0296  0.00483 | 0.0349  **0.0176**  **0.0167**  **0.0184**  0.0567  0.0261  0.0191  0.0168  0.0166  0.0166  0.0166  0.0166  0.0166 | 0.637  **2.01x10^-5^**  **6.68x10^-7^**  **5.02x10^-28^**  0.371  0.277  0.428  0.343  0.207  0.524  0.227  0.0745  0.771 | -  0.0340  -0.0780  0.265  0.137  -0.0361  -0.0479  0.0185  0.00609  -0.00533  0.0281  -0.0143  0.0176 | -  0.0249  0.0231  0.0248  0.0782  0.0355  0.0263  0.0231  0.0230  0.0230  0.0229  0.0230  0.0230 | -  0.171  **7.44x10^-4^**  **8.00x10^-26^**  0.0801  0.309  0.0687  0.423  0.791  0.817  0.220  0.535  0.444 | -  **0.119**  **-0.0892**  **0.102**  -0.0610  -0.0141  0.0214  0.0103  0.0383  -0.0162  0.0101  -0.0454  -0.0173 | -  **0.0250**  **0.0243**  **0.0249**  0.0827  0.0386  0.0280  0.0244  0.0241  0.0241  0.0242  0.0241  0.0241 | -  **2.18x10^-6^**  **2.46x10^-4^**  **4.80x10^-5^**  0.461  0.715  0.446  0.672  0.112  0.501  0.675  0.0590  0.472 |
| Null model + parental style | | 0.0392 | 0.0167 | 0.0193 | 0.0434 | 0.0231 | 0.0601 | 0.0371 | 0.0242 | 0.126 |
| Null model **+ BMI PRS** | | **0.199** | **0.0163** | **6.64x10^-34^** | **0.180** | **0.0226** | **2.37x10^-15^** | **0.222** | **0.0235** | **1.10x10^-20^** |
| Null model + Parental style **+ BMI PRS** | | 0.0375  **0.199** | 0.0164  **0.0162** | 0.0221  **7.61x10^-34^** | 0.0429  **0.180** | 0.0227  **0.0226** | 0.0588  **2.36x10^-15^** | 0.0339  **0.222** | 0.0236  **0.0235** | 0.151  **1.30x10^-20^** |
| Null model + Parental style x BMI PRS | | 0.00541 | 0.0173 | 0.755 | -0.00834 | 0.0241 | 0.729 | 0.0302 | 0.0253 | 0.233 |

Supplementary Table S6a: Effects of adding variables and interactions to the null model (uppermost line) predicting variance in untransformed BMI at 11 years old, with parenting as the environment of interest. Significant (p<0.00417) terms are in bold. Interactions include all main effects, covariates and covariate interaction terms [^17^](#_ENREF_17).

Supplementary Table S6b

| **Untransformed BMI at 11 years old, with socioeconomic status** | | | | | | | | | | |
| --- | --- | --- | --- | --- | --- | --- | --- | --- | --- | --- |
|  | | **Full cohort (N = 3414)** | | | **Females (N = 1750)** | | | **Males (N = 1664)** | | |
| **Coefficient** | | **B** | **SE** | ***p*** | **B** | **SE** | ***p*** | **B** | **SE** | ***p*** |
| Null Model | Sex  **Age** Parenting  **Pubertal development**  Wave  PC1  PC2  PC3  PC4  PC5  PC6  PC7  PC8 | 0.00903  **0.0829**  0.0469  **0.205**  0.0488  -0.0301  -0.0136  0.00997  0.0216  -0.0106  0.0185  -0.0306  0.00353 | 0.0352  **0.0176**  0.0167  **0.0184**  0.0568  0.0261  0.0192  0.0168  0.0166  0.0166  0.0166  0.0166  0.0166 | 0.797  **2.68x10^-6^**  0.00511  **2.12x10^-28^**  0.390  0.250  0.479  0.552  0.193  0.523  0.265  0.0662  0.832 | -  0.0397  0.0515  **0.268**  0.128  -0.0338  -0.0433  0.0136  0.00788  -0.00434  0.0289  -0.0154  0.0169 | -  0.0249  0.0230  **0.0248**  0.0783  0.0355  0.0263  0.0231  0.0230  0.0230  0.0230  0.0230  0.0230 | -  0.111  0.0251  **2.23x10^-26^**  0.103  0.342  0.100  0.558  0.732  0.850  0.209  0.505  0.464 | -  **0.129**  0.0441  **0.102**  -0.0547  -0.0210  0.0192  0.00308  0.0379  -0.0179  0.00617  -0.0459  -0.0194 | -  **0.0250**  0.0242  **0.0250**  0.0830  0.0387  0.0281  0.0244  0.0242  0.0241  0.0242  0.0241  0.0242 | -  **3.01x10^-7^**  0.0689  **4.99x10^-5^**  0.510  0.588  0.494  0.899  0.117  0.458  0.799  0.0572  0.423 |
| Null model **+ SES** | | **-0.0793** | **0.0168** | **2.30x10^-6^** | **-0.0731** | **0.0232** | **0.00167** | **-0.0861** | **0.243** | **4.13x10^-4^** |
| Null model **+ BMI PRS** | | **0.201** | **0.0163** | **3.27x10^-34^** | **0.181** | **0.0226** | **1.86x10^-15^** | **0.224** | **0.0235** | **7.01x10^-21^** |
| Null model **+ SES + BMI PRS** | | **-0.0747**  **0.199** | **0.0164**  **0.0162** | **5.40x10^-6^**  **7.61x10^-34^** | **-0.0701**  **0.180** | **0.0228**  **0.0226** | **0.00212**  **2.36x10^-15^** | **-0.0799**  **0.222** | **0.0237**  **0.0235** | **7.78x10^-4^**  **1.30x10^-20^** |
| Null model + SES x BMI PRS | | -0.0335 | 0.0165 | 0.0428 | -0.0262 | 0.0235 | 0.265 | -0.0335 | 0.0240 | 0.162 |

Supplementary Table S6b: Effects of adding variables and interactions to the null model (uppermost line) predicting variance in untransformed BMI at 11 years old, with socioeconomic status as the environment of interest. Significant (p<0.00417) terms are in bold. Interactions include all main effects, covariates and covariate interaction terms [^17^](#_ENREF_17).

Supplementary Table S7a

| **Untransformed BMI change across adolescence, with parenting** | | | | | | | | | | |
| --- | --- | --- | --- | --- | --- | --- | --- | --- | --- | --- |
|  | | **Full cohort (N = 1943)** | | | **Females (N = 1043)** | | | **Males (N = 900)** | | |
| **Coefficient** | | **B** | **SE** | ***p*** | **B** | **SE** | ***p*** | **B** | **SE** | ***p*** |
| Null Model | **Untransformed BMI at 11**  Sex  Age SES  Pubertal development  Wave  PC1  PC2  PC3  **PC4**  PC5  PC6  **PC7**  PC8 | **-0.546**  -0.0375  0.0179  0.00631  0.0117  -0.0127  0.0343  -0.0209  0.0122  **-0.0588**  -0.00851  0.0107  **-0.0677**  0.0102 | **0.0198**  0.0405  0.0202  0.0193  0.0214  0.0656  0.0299  0.0221  0.0194  **0.0191**  0.0191  0.0191  **0.0191**  0.0191 | **5.23x10^-142^**  0.354  0.377  0.744  0.587  0.846  0.251  0.343  0.529  **0.00209**  0.656  0.576  **3.95x10^-4^**  0.593 | **-0.572**  -  -0.0139  -0.0179  0.0189  0.0714  -0.00368  -0.0107  -0.00568  -0.0573  -0.0214  0.0167  -0.0607  0.00453 | **0.0268**  -  0.0280  0.0259  0.0286  0.0881  0.0395  0.0297  0.0260  0.0258  0.0257  0.0257  0.0257  0.0257 | **5.78x10^-84^**  -  0.620  0.490  0.509  0.419  0.926  0.720  0.827  0.0263  0.407  0.516  0.0186  0.860 | **-0.511**  -  0.0551  0.0368  0.0116  -0.125  0.0837  -0.0332  0.0313  -0.0563  0.00433  -0.00229  -0.0714  0.0207 | **0.0295**  -  0.0297  0.0293  0.0297  0.0988  0.0459  0.0334  0.0293  0.0289  0.0287  0.0289  0.0288  0.0288 | **5.99x10^-58^**  -  0.0643  0.209  0.696  0.205  0.0685  0.321  0.287  0.0517  0.881  0.937  0.0133  0.472 |
| Null model + parental style | | -0.00596 | 0.0194 | 0.758 | -0.0101 | 0.0260 | 0.697 | 1.22x10^‑4^ | 0.0290 | 0.997 |
| **Null model + BMI PRS** | | **0.0855** | **0.0194** | **1.07x10^-5^** | **0.0979** | **0.0259** | **1.70x10^-4^** | 0.0724 | 0.0293 | 0.0137 |
| Null model + parental style  **+ BMI PRS** | | -0.00715  **0.0856** | 0.0193  **0.0194** | 0.711  **1.05x10^-5^** | -0.0113  **0.0980** | 0.0258  **0.0259** | 0.661  **1.67x10^-4^** | -0.00113  0.0725 | 0.0289  0.0293 | 0.969  0.0137 |
| Null model + Parental style x BMI PRS | | -0.00150 | 0.0204 | 0.941 | 0.0166 | 0.0278 | 0.550 | -0.0403 | 0.0307 | 0.190 |

Supplementary Table S7a: Effects of adding variables and interactions to the null model (uppermost line) predicting change in BMI across adolescence, with parenting as the environment of interest. Significant (p<0.00417) terms are in bold. Interactions include all main effects, covariates and covariate interaction terms [^17^](#_ENREF_17).

Supplementary Table S7b

| **BMI change across adolescence, with socioeconomic status** | | | | | | | | | | |
| --- | --- | --- | --- | --- | --- | --- | --- | --- | --- | --- |
|  | | **Full cohort (N = 1943)** | | | **Females (N = 1043)** | | | **Males (N = 900)** | | |
| **Coefficient** | | **B** | **SE** | ***p*** | **B** | **SE** | ***P*** | **B** | **SE** | ***p*** |
| Null Model | **Untransformed BMI at 11**  Sex  Age Parental style  Pubertal development  Wave  PC1  PC2  PC3  **PC4**  PC5  PC6  **PC7**  PC8 | **-0.546**  -0.0363  0.0169  -0.00644  0.0116  -0.0119  0.0342  -0.0212  0.0126  **-0.0588**  -0.00836  0.0108  **-0.0676**  0.00996 | **0.0197**  0.0407  0.0203  0.0193  0.0214  0.0655  0.0299  0.0221  0.0193  **0.0191**  0.0191  0.0191  **0.0191**  0.0191 | **9.50x10^-143^**  0.372  0.407  0.738  0.590  0.857  0.252  0.337  0.517  **0.00210**  0.661  0.570  **4.05x10^-4^**  0.602 | **-0.569**  -  -0.0139  -0.00823  0.0190  0.0704  -0.00360  -0.00987  -0.00642  -0.0570  -0.0208  0.0162  -0.0612  0.00511 | **0.0268**  -  0.0280  0.0258  0.0286  0.0882  0.0396  0.0297  0.0260  0.0258  0.0258  0.0257  0.0257  0.0257 | **1.54x10^-83^**  -  0.619  0.750  0.507  0.425  0.928  0.740  0.805  0.0271  0.420  0.528  0.0176  0.842 | **-0.515**  -  0.0524  -0.00215  0.00951  -0.126  0.0859  -0.0333  0.0347  -0.0548  0.00553  -4.50x10^-4^  -0.0715  0.0199 | **0.0294**  -  0.0299  0.0290  0.0297  0.0989  0.0459  0.0335  0.0293  0.0289  0.0288  0.0289  0.0288  0.0288 | **3.64x10^-59^**  -  0.0805  0.941  0.749  0.205  0.0618  0.319  0.237  0.0582  0.847  0.988  0.0133  0.490 |
| Null model + SES | | 0.00581 | 0.0194 | 0.764 | -0.0189 | 0.0260 | 0.468 | 0.0368 | 0.0294 | 0.210 |
| **Null model + BMI PRS** | | **0.0857** | **0.0194** | **1.03x10^-5^** | **0.0977** | **0.0259** | **1.75x10^-4^** | 0.0726 | 0.0294 | 0.0136 |
| Null model + SES  **+ BMI PRS** | | 0.00498  **0.0856** | 0.0193  **0.0194** | 0.796  **1.05x10^-5^** | -0.0204  **0.0980** | 0.0258  **0.0259** | 0.431  **1.67x10^-4^** | 0.0366  0.0725 | 0.0293  0.0293 | 0.211  0.0137 |
| Null model + SES x BMI PRS | | -0.0448 | 0.0201 | 0.0263 | -0.0671 | 0.0274 | 0.0146 | -0.00940 | 0.0310 | 0.762 |

Supplementary Table S7b: Effects of adding variables and interactions to the null model (uppermost line) predicting change in BMI across adolescence, with socioeconomic status as the environment of interest. Significant (p<0.00417) terms are in bold. Interactions include all main effects, covariates and covariate interaction terms [^17^](#_ENREF_17).

Supplementary Table S8

| **Coefficient** | **B** | **SE** | ***p*** | **Adjusted R^2^** |
| --- | --- | --- | --- | --- |
| **BMI at 11 years old, with parenting** | | | | |
| Null model | Supplementary Table 7a | | | 0.0637 |
| (Null model) + Parental style | 0.0392 | 0.0167 | 0.0193 | 0.0649 |
| (Null model) **+ rs9939609** | **0.145** | **0.0240** | **1.48x10^-9^** | 0.0733 |
| (Null model) + Parental style  **+ rs9939609** | 0.0387  **0.145** | 0.0167  **0.0240** | 0.0202  **1.69x10^-9^** | 0.0746 |
| (Null model) + Parental style x rs9939609 | -0.0197 | 0.0245 | 0.422 | 0.0763 |
| **BMI at 11 years old, with SES** | | | | |
| Null model | Supplementary Table 7b | | | 0.0590 |
| Null model **+ SES** | **-0.0793** | **0.0168** | **2.30x10^-6^** | 0.0649 |
| Null model **+ rs9939609** | **0.145** | **0.0240** | **1.97x10^-9^** | 0.0689 |
| Null model **+** **SES** **+ rs9939609** | **-0.0779**  **0.145** | **0.0167**  **0.0240** | **3.20x10^-6^**  **1.69x10^-9^** | 0.0745 |
| Null model + SES x rs9939609 | -0.0219 | 0.0243 | 0.366 | 0.0756 |

Supplementary Table S8: Effects of adding variables and interactions to the null model predicting variance in unrtransformed BMI at 11 years old, with parenting as the environment of interest and FTO variant rs9939609 as the genotype of interest. Significant (p<0.00417) terms are in bold. Interactions include all main effects, covariates and covariate interaction terms [^17^](#_ENREF_17).

Supplementary Table S9

| **Coefficient** | **B** | **SE** | ***p*** | **Adjusted R^2^** |
| --- | --- | --- | --- | --- |
| **BMI change, with parenting** | | | | |
| Null model | Supplementary Table 3a | | | 0.296 |
| (Null model) + Parental style | -0.00596 | 0.0194 | 0.758 | 0.296 |
| (Null model) + rs9939609 | 0.0419 | 0.0280 | 0.135 | 0.297 |
| (Null model) + Parental style  + rs9939609 | -0.00453  0.0420 | 0.0193  0.0281 | 0.815  0.134 | 0.297 |
| (Null model) + Parental style x rs9939609 | 0.00373 | 0.0285 | 0.896 | 0.295 |
| **BMI change, with SES** | | | | |
| Null model | Supplementary Table 3b | | | 0.296 |
| Null model + SES | 0.00581 | 0.0194 | 0.764 | 0.296 |
| Null model + rs9939609 | 0.0422 | 0.0280 | 0.133 | 0.297 |
| Null model + SES + rs9939609 | 0.00728  0.0420 | 0.0193  0.0281 | 0.707  0.134 | 0.297 |
| Null model + SES x rs9939609 | 0.0213 | 0.0289 | 0.460 | 0.299 |

Supplementary Table S9: Effects of adding variables and interactions to the null model predicting variance in unrtransformed BMI at 11 years old, with SES as the environment of interest and FTO variant rs9939609 as the genotype of interest. Significant (p<0.00417) terms are in bold. Interactions include all main effects, covariates and covariate interaction terms [^17^](#_ENREF_17).

Supplementary Table S10a

| **BMI at 11 years old, with parenting** | | | | | | | | | | |
| --- | --- | --- | --- | --- | --- | --- | --- | --- | --- | --- |
|  | | **Full cohort (N = 3414)** | | | **Females (N = 1750)** | | | **Males (N = 1664)** | | |
| **Coefficient** | | **B** | **SE** | ***p*** | **B** | **SE** | ***p*** | **B** | **SE** | ***p*** |
| Null Model | Sex  **Age SES**  **Pubertal development**  Wave  PC1  PC2  PC3  PC4  PC5  PC6  PC7  PC8 | 0.0260  **0.0795**  **-0.0766**  **0.211**  0.0598  -0.0312  -0.00982  0.0149  0.0193  -0.0101  0.0169  -0.0329  0.00575 | 0.0349  **0.0176**  **0.0167**  **0.0183**  0.0566  0.0260  0.0191  0.0167  0.0166  0.0166  0.0166  0.0166  0.0166 | 0.457  **6.12x10^-6^**  **4.42x10^-6^**  **5.44x10^-30^**  0.291  0.230  0.607  0.372  0.245  0.541  0.307  0.0472  0.728 | -  0.0346  -0.0740  0.276  0.151  -0.0443  -0.0429  0.0190  0.00107  -0.00566  0.0246  -0.0190  0.0181 | -  0.0248  0.0230  0.0247  0.0780  0.0353  0.0262  0.0231  0.0229  0.0229  0.0229  0.0229  0.0229 | -  0.162  **0.00131**  **4.38x10^-28^**  0.0532  0.211  0.102  0.410  0.963  0.805  0.281  0.407  0.431 | -  **0.126**  **-0.0796**  **0.106**  -0.0566  -0.0107  0.0267  0.00822  0.0402  -0.0148  0.00705  -0.0461  -0.0155 | -  **0.0249**  **0.0243**  **0.0249**  0.0826  0.0386  0.0280  0.0244  0.0241  0.0241  0.0241  0.0240  0.0241 | -  **4.90x10^-7^**  **0.00106**  **2.30x10^-5^**  0.493  0.782  0.340  0.736  0.0954  0.538  0.770  0.0553  0.520 |
| Null model + parental style | | 0.0378 | 0.0167 | 0.0239 | 0.0413 | 0.0230 | 0.0730 | 0.0364 | 0.0242 | 0.133 |
| Null model **+ BMI PRS_PT_1** | | **0.174** | **0.0164** | **5.58x10^-26^** | **0.145** | **0.0227** | **2.32x10^-10^** | **0.207** | **0.0238** | **7.93x10^-18^** |
| Null model + Parental style **+ BMI PRS_PT_1** | | 0.0370  **0.174** | 0.0165  **0.0164** | 0.0248  **5.84x10^-26^** | 0.0379  **0.144** | 0.0227  **0.0227** | 0.0956  **2.93x10^-10^** | 0.0395  **0.208** | 0.0237  **0.0238** | 0.0951  **6.21x10^-18^** |
| Null model + Parental style x BMI PRS_PT_1 | | 0.00397 | 0.0168 | 0.814 | 0.0346 | 0.0246 | 0.159 | -0.0168 | 0.0238 | 0.481 |

Supplementary Table S10a: Effects of adding variables and interactions to the null model (uppermost line) predicting variance in BMI at 11 years old, with parenting as the environment of interest and a PRS made up of all SNPs. Significant (p<0.00417) terms are in bold. Interactions include all main effects, covariates and covariate interaction terms [^17^](#_ENREF_17).

Supplementary Table S10b

| **BMI at 11 years old, with socioeconomic status** | | | | | | | | | | |
| --- | --- | --- | --- | --- | --- | --- | --- | --- | --- | --- |
|  | | **Full cohort (N = 3414)** | | | **Females (N = 1750)** | | | **Males (N = 1664)** | | |
| **Coefficient** | | **B** | **SE** | ***p*** | **B** | **SE** | ***p*** | **B** | **SE** | ***p*** |
| Null Model | Sex  **Age** Parenting  **Pubertal development**  Wave  PC1  PC2  PC3  PC4  PC5  PC6  PC7  PC8 | 0.0187  **0.0868**  0.0448  **0.212**  0.0581  -0.0329  -0.00838  0.00950  0.0199  -0.0102  0.0156  -0.0338  0.00455 | 0.0351  **0.0176**  0.0167  **0.0184**  0.0567  0.0261  0.0191  0.0167  0.0166  0.0166  0.0166  0.0166  0.0166 | 0.594  **8.39x10^-7^**  0.00726  **2.43x10^-30^**  0.306  0.207  0.661  0.57  0.229  0.538  0.349  0.0418  0.784 | -  0.0401  0.0490  **0.279**  0.142  -0.0421  -0.0384  0.0143  0.00277  -0.00473  0.0254  -0.0200  0.0174 | -  0.0248  0.0229  **0.0247**  0.0781  0.0354  0.0262  0.0231  0.0229  0.0229  0.0229  0.0229  0.0230 | -  0.107  0.0326  **1.22x10^-28^**  0.0687  0.235  0.143  0.536  0.904  0.837  0.268  0.382  0.449 | -  **0.135**  0.0426  **0.106**  -0.0507  -0.0170  0.0247  0.00178  0.0399  -0.0164  0.00354  -0.0466  -0.0173 | -  **0.0250**  0.0242  **0.0250**  0.0829  0.0387  0.0280  0.0243  0.0242  0.0241  0.0242  0.0241  0.0241 | -  **7.18x10^-8^**  0.0784  **2.46x10^-5^**  0.540  0.660  0.379  0.942  0.0991  0.497  0.884  0.0532  0.473 |
| Null model **+ SES** | | **-0.0730** | **0.0167** | **1.33x10^-5^** | **-0.0694** | **0.0231** | **0.00274** | **-0.0766** | **0.243** | **0.00167** |
| Null model **+ BMI PRS_PT_1** | | **0.177** | **0.0164** | **1.14x10^-26^** | **0.145** | **0.0228** | **2.56x10^-10^** | **0.212** | **0.0238** | **1.12x10^-18^** |
| Null model **+ SES + BMI PRS_PT_1** | | **-0.0655**  **0.174** | **0.0165**  **0.0164** | **7.21x10^-5^**  **5.85x10^-26^** | **-0.0677**  **0.144** | **0.0229**  **0.0227** | **0.00313**  **2.93x10^-10^** | **-0.0611**  **0.208** | **0.0239**  **0.0238** | **0.0106**  **6.21x10^-18^** |
| Null model + SES x BMI PRS_PT_1 | | -0.0398 | 0.0168 | 0.0178 | -0.0398 | 0.0232 | 0.0863 | -0.0240 | 0.0249 | 0.335 |

Supplementary Table S10b: Effects of adding variables and interactions to the null model (uppermost line) predicting variance in BMI at 11 years old, with socioeconomic status as the environment of interest and a PRS made up of all SNPs. Significant (p<0.00417) terms are in bold. Interactions include all main effects, covariates and covariate interaction terms [^17^](#_ENREF_17).

Supplementary Table S11a

| **BMI change across adolescence, with parenting** | | | | | | | | | | |
| --- | --- | --- | --- | --- | --- | --- | --- | --- | --- | --- |
|  | | **Full cohort (N = 1943)** | | | **Females (N = 1043)** | | | **Males (N = 900)** | | |
| **Coefficient** | | **B** | **SE** | ***p*** | **B** | **SE** | ***p*** | **B** | **SE** | ***p*** |
| Null Model | **BMI at 11**  Sex  Age SES  Pubertal development  Wave  PC1  PC2  PC3  **PC4**  PC5  PC6  **PC7**  PC8 | **-0.528**  -0.0281  0.0178  0.0113  0.0126  -0.0135  0.0353  -0.0157  0.0105  **-0.0588**  -0.00878  0.00908  **-0.0710**  0.00997 | **0.0201**  0.0410  0.0205  0.0196  0.0218  0.0665  0.0303  0.0224  0.0197  **0.0193**  0.0193  0.0193  **0.0194**  0.0193 | **1.56x10^-130^**  0.493  0.386  0.565  0.562  0.839  0.244  0.483  0.594  **0.00242**  0.650  0.639  **0.000251**  0.606 | **-0.546**  -  -0.0138  -0.0120  0.0206  0.0749  -0.00625  -0.00365  -0.00693  -0.0593  -0.0234  0.0134  -0.0666  0.00450 | **0.0275**  -  0.0286  0.0264  0.0293  0.0901  0.0404  0.0303  0.0266  0.0263  0.0263  0.0262  0.0263  0.0262 | **1.56x10^-74^**  -  0.628  0.650  0.482  0.406  0.877  0.904  0.794  0.0243  0.373  0.611  0.0114  0.864 | **-0.505**  -  0.0547  0.0407  0.0128  -0.130  0.0890  -0.0306  0.0290  -0.0536  0.00648  -0.00208  -0.0708  0.0207 | **0.0296**  -  0.0298  0.0294  0.0298  0.0992  0.0461  0.0335  0.0295  0.0290  0.0288  0.0290  0.0289  0.0289 | **1.55x10^-56^**  -  0.0673  0.167  0.669  0.190  0.0536  0.362  0.326  0.0650  0.822  0.943  0.0144  0.473 |
| Null model + parental style | | -0.00848 | 0.0196 | 0.666 | -0.0147 | 0.0265 | 0.580 | -9.79x10^‑5^ | 0.0291 | 0.997 |
| **Null model + BMI PRS_PT_1** | | **0.0977** | **0.0196** | **7.16x10^-7^** | **0.111** | **0.0265** | **3.12x10^-5^** | 0.0847 | 0.0296 | 0.00433 |
| Null model + parental style  **+ BMI PRS_PT_1** | | -0.00976  **0.0978** | 0.0195  **0.0196** | 0.617  **6.97x10^-7^** | -0.0172  **0.111** | 0.0263  **0.0265** | 0.514  **2.96x10^-5^** | -0.000319  0.0847 | 0.0290  0.0296 | 0.991  0.00435 |
| Null model + Parental style x BMI PRS_PT_1 | | 0.00817 | 0.0207 | 0.694 | 0.00894 | 0.0292 | 0.760 | -0.00927 | 0.0306 | 0.762 |

Supplementary Table S11a: Effects of adding variables and interactions to the null model (uppermost line) predicting change in BMI across adolescence, with parenting as the environment of interest and a PRS made up of all SNPs. Significant (p<0.00417) terms are in bold. Interactions include all main effects, covariates and covariate interaction terms [^17^](#_ENREF_17).

Supplementary Table S11b

| **BMI change across adolescence, with socioeconomic status** | | | | | | | | | | |
| --- | --- | --- | --- | --- | --- | --- | --- | --- | --- | --- |
|  | | **Full cohort (N = 1943)** | | | **Females (N = 1043)** | | | **Males (N = 900)** | | |
| **Coefficient** | | **B** | **SE** | ***p*** | **B** | **SE** | ***P*** | **B** | **SE** | ***p*** |
| Null Model | **BMI at 11**  Sex  Age Parental style  Pubertal development  Wave  PC1  PC2  PC3  **PC4**  PC5  PC6  **PC7**  PC8 | **-0.528**  -0.0265  0.0162  -0.00936  0.0125  -0.0121  0.0351  -0.0161  0.0111  **-0.0587**  -0.00854  0.00938  **-0.0708**  0.00955 | **0.0200**  0.0412  0.0206  0.0196  0.0218  0.0664  0.0303  0.0224  0.0196  **0.0193**  0.0193  0.0193  **0.0194**  0.0193 | **2.99x10^-131^**  0.521  0.432  0.632  0.569  0.855  0.246  0.470  0.572  **0.00246**  0.659  0.628  **0.000262**  0.621 | **-0.544**  -  -0.0146  -0.0133  0.0206  0.0754  -0.00668  -0.00342  -0.00733  -0.0591  -0.0228  0.0128  -0.0670  0.00486 | **0.0275**  -  0.0286  0.0264  0.0293  0.0901  0.0404  0.0303  0.0265  0.0263  0.0263  0.0262  0.0263  0.0262 | **3.36x10^-74^**  -  0.611  0.613  0.484  0.403  0.869  0.910  0.783  0.0249  0.387  0.624  0.0109  0.853 | **-0.509**  -  0.0516  -0.00262  0.0104  -0.131  0.0915  -0.0307  0.0326  -0.0520  0.00784  -4.49x10^-5^  -0.0709  0.0198 | **0.0295**  -  0.0301  0.0291  0.0299  0.0993  0.0461  0.0336  0.0294  0.0290  0.0289  0.0290  0.0289  0.0289 | **1.11x10^-57^**  -  0.0862  0.928  0.727  0.189  0.0475  0.360  0.267  0.0738  0.786  0.999  0.0144  0.493 |
| Null model + SES | | 0.0106 | 0.0196 | 0.591 | -0.0135 | 0.0265 | 0.612 | 0.0407 | 0.0295 | 0.168 |
| **Null model + BMI PRS_PT_1** | | **0.0975** | **0.0196** | **7.45x10^-7^** | **0.111** | **0.0265** | **2.94x10^-5^** | 0.0824 | 0.0296 | 0.00550 |
| Null model + SES  **+ BMI PRS_PT_1** | | 0.0128  **0.0978** | 0.0195  **0.0196** | 0.512  **6.97x10^-7^** | -0.0135  **0.111** | 0.0263  **0.0265** | 0.609  **2.96x10^-5^** | 0.0449  0.0847 | 0.0294  0.0296 | 0.127  0.00435 |
| Null model + SES x BMI PRS_PT_1 | | -0.0294 | 0.0203 | 0.147 | -0.00776 | 0.0273 | 0.777 | -0.0574 | 0.0314 | 0.0684 |

Supplementary Table S11b: Effects of adding variables and interactions to the null model (uppermost line) predicting change in BMI across adolescence, with socioeconomic status as the environment of interest and a PRS made up of all SNPs. Significant (p<0.00417) terms are in bold. Interactions include all main effects, covariates and covariate interaction terms [^17^](#_ENREF_17).
